# Effect of Differential Speed Rotation Technology on the Forming Uniformity in Flexible Rolling Process

**DOI:** 10.3390/ma11101906

**Published:** 2018-10-08

**Authors:** Yi Li, Mingzhe Li, Kai Liu, Zhuo Li

**Affiliations:** Dieless Forming Technology Center, Jilin University, Changchun 130025, China; liyi6929@163.com (Y.L.); liukaijlu@163.com (K.L.); zli16@mails.jlu.edu.cn (Z.L.)

**Keywords:** flexible rolling, convex surface part, numerical simulation, differential speed rotation, forming uniformity

## Abstract

As the local forming non-uniform of the formed curved surface part with larger bending deformation is the one of common defects, the utilization ratio of metal plate greatly reduces due to this defect, and cost of production is also increasing. In this paper, the differential speed rotation technology of flexible rolling process was proposed firstly to solve this forming defect. The finite element model was established, the reason of the local forming non-uniform was discussed; the effect of differential speed rotation technology on the forming uniform was studied. The results show that: Flexible rolling is a process based on thickness reduction, in this forming process, the thickness reduces sharply near the back end of metal plate, the local forming non-uniform of formed curved surface part is caused during this process; the differential speed rotation technology is applied in flexible rolling, with increasing rotation speed difference between upper and lower roll set, the forming uniformity of the formed curved surface part is greatly improved. The results of numerical simulation are in agreement with the result of forming experiments.

## 1. Introduction

Three-dimensional surface parts with various bending radius have been used in many fields, they become necessities in the modern manufacturing industry. As the traditional forming processes aren’t suitable for the higher demands of market [1,2], the forming processes with more efficiency, and lower cost have received the most attention. In the earlier stage, YAMASHITA proposed the forming apparatus for double curvature surface part, the forming tool with the discrete linear contour was employed in the forming process. However, the formed parts were poor-quality due to the insufficient forming continuity [3]. Such as rolling and roll-bending [4,5], continuous forming processes have the advantages of highly productivity and low cost, because dedicated dies and time consuming equipment operations are unnecessary in these processes. As continuous forming processes combine both the continuous forming concept and flexible forming idea, the formed parts with various sizes and shapes are obtained. Three upper and lower roll sets were employed in the line array roll set process developed by Yoon et al., and forming roll sets consisted of discrete linear rollers; the transversal bending of formed part is achieved by the contour of roll sets; the longitude bending of forming part is achieved by three-point bending [6,7,8]. When the forming tools are straight rigid roll sets, the shapes of formed parts are only cylindrical or conical in traditional roll-bending [9,10]. Flexible rolling process is the combination of rolling and multi-points process, two forming roll sets were employed in flexible rolling process, the double bending is achieved by the non-uniform roll gap. A comparison with previous forming process showed that only two forming roll sets were employed in this process, the forming system is more simplified [11,12]. The forming formation was proposed in flexible rolling process, the parametric equations were derived, and the computing method of roll gap for bending deformation was presented [13,14]. In this study, as the local forming non-uniform of the formed curved surface part with larger bending deformation is the one of common defects, the reason of this defect was studied in flexible rolling; the differential speed rotation technology of flexible rolling process was proposed, by increasing the rotation speed difference between two forming roll sets, the forming uniformity of the formed curved surface part is greatly improved.

## 2. Materials and Methods

### 2.1. Forming Process

**Figure 1:**
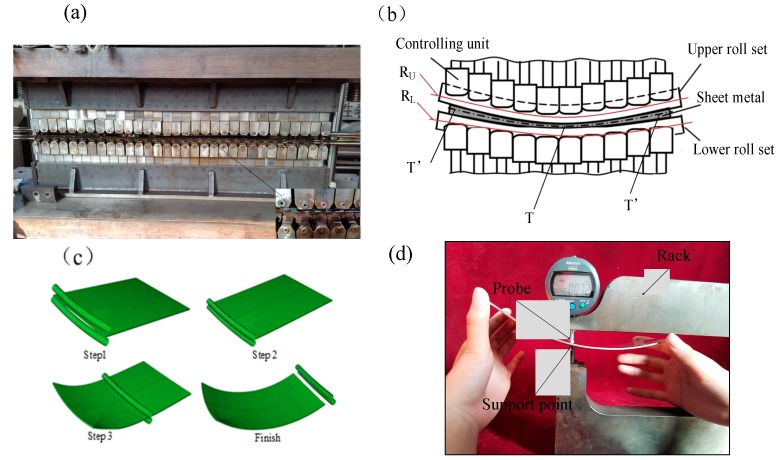
Flexible rolling apparatus (**a**) forming equipment (**b**) the schematic (**c**) forming process (**d**) the measuring instrument for thickness.

The flexible rolling experimental equipment is developed by Jilin University (Figure 1a). The schematic of experimental equipment is presented in Figure 1b. A pair of forming roll sets is employed in this apparatus, they are arranged up and down, and the contour of each forming roll set can be adjusted into a specific curve by moving each controlling unit up and down. In this process, the contours of forming roll sets are respectively similar to two different radius arcs. *R_U_* is defined as the upper roll set contour radius, and *R_L_* is defined as the lower roll set contour radius when the formed part is convex surface part, *R_U_* < *R_L_*. After processing, due to the shape of roll gap, the thickness distribution of formed part is increasing from the center to both sides (*T* is defined as the minimum thickness; *T’* is defined as the maximum thickness). The forming process of the convex surface part is shown in Figure 1c. Before processing, the contour of each roll set should be adjusted into the target radius. First, the lower forming roll set is immobile; the upper forming roll set drops to the target position, the metal plate is caught. Then, the forming roll sets begin to rotate about their own center axis with same speed and opposite rotation direction, a metal plate is fed under the friction; the metal plate is continuously deforming with thickness reduction, strength properties of the material increase, and with increasing rolling reduction, the thickness reduction is compensated as working hardening, the convex surface part is formed finally during this process. The measuring tool is the digital instrument with a measuring probe, the metrological characteristic of digital instrument is shown in Table 1. The digital instrument is fixed in the rack, this probe can move up and down, as shown in Figure 1d, before measuring, the digital instrument must be set to zero by standard gauge block with thickness 1.8 mm, when the distance between measuring point and support point is 1.8 mm, the data is 0 mm in the display, so the data is the thickness reduction of metal plate compared to the initial thickness (1.8 mm) in the display, picking equidistant measuring points in the measuring line of formed part; the steps of measuring points are from the front end to the back end of formed part in the longitude direction and from left to right in the transversal direction. In the measuring process, the formed part must be steadily fixed in the support point and the measuring point must be clamped vertically by probe and support point, as the three-dimensional bending deformation is larger, mechanical fixture is not flexible enough to vertically measure all the points, so the hand holder is more suitable to measure the thickness of formed surface part with larger deformation, and measurement precision is satisfied by averaging the repeated testing values. The measuring probe of the digital instrument is pressing down to the upper surface of the formed part, the distance between the probe and support point is the thickness of the formed surface part and the corresponding thickness reduction is displayed in the measuring tool, the thickness reduction is 0.072 mm (in Figure 1d), after measuring the thickness reduction of each point, the thickness distribution in the measuring line is obtained.

### 2.2. The Formation Mechanism of the Convex Surface Part

The formation mechanism of the convex surface part is presented as follows. *v* is defined as the transversal direction, *u* is defined as the longitude direction. In flexible rolling, the metal plate is thinned and the corresponding length elongated in the longitude direction. At different points, the elongation of longitudinal fibers is varied as the non-uniform roll gap. At the point *v*, the length is Δ*l*, so the longitude elongation is *dε_u_*(*v*) = *d*(Δ*l*)/Δ*l*. So the total strain is:

(1) $$\varepsilon_{u}(v)=\int_{\Delta l_{0}}^{\Delta l}\frac{d(\Delta l)}{\Delta l}=\ln\frac{\Delta l(v)}{\Delta l_{0}}$$

where Δ*l*_0_ is the initial longitude length, Δ*l*(*v*) is the length at point *v* after deformation.

After processing, at the point *v*, the longitude length is:

(2) $$\Delta l(v)=\Delta l_{0}\;\exp[\varepsilon_{u}(v)]$$

**Figure 2:**
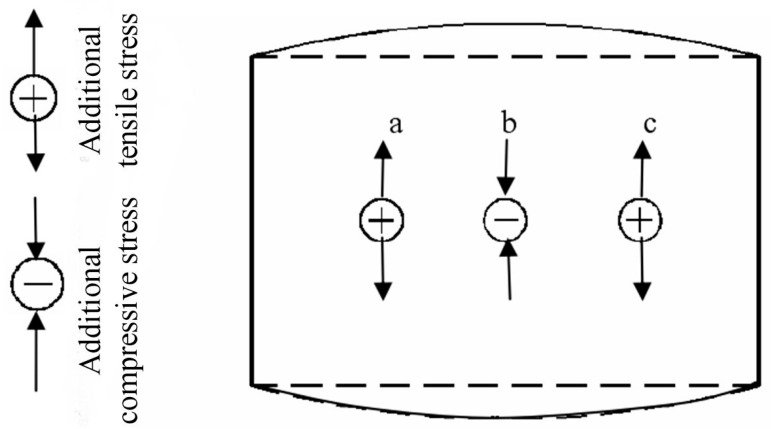
The schematic of longitude bending.

As the transversal thickness of metal plate is unevenly thinned due to the roll gap, so the transversal bending is similar to roll gap shape; because of the varied thickness reduction, the longitude elongation is varied along the transversal direction, the part with a large longitudinal elongation is restrained by the adjacent part with a small elongation, additional compressive stress is obtained (b in Figure 2); while the part with a small longitudinal elongation is stretched by the adjacent part with a large elongation, additional tensile stress is obtained (a and c in Figure 2). As a result, the longitude bending of forming convex surface part is achieved.

After processing, the longitudinal bending deformation in the cross-section can be expressed by longitudinal elongation at any point. Longitude curvature: *ρ_u_*^−1^ = *dl*/*dθ*. Where *θ* is the normal angel of the longitude curve *p*(*u*). So the elongation of longitude fiber is:

(3) $$\Delta l(v)=\int_{0}^{\Delta \theta }\rho_{u}d\theta \approx \rho_{u}(v)\Delta \theta (v)$$

where *ρ_u_*(*v*) is average longitudinal curvature radius, Δ*θ* is the normal angle of surface which corresponds to the length Δ*l*.

*ρ_u_* can be expressed as follow:

(4) $$\rho_{u}(v)=\frac{\Delta l_{0}}{\Delta \theta (v)}\;\exp[\varepsilon_{u}(v)]$$

So the longitudinal curve of the processed surface can be obtained.

### 2.3. Finite Element Model

**Figure 3:**
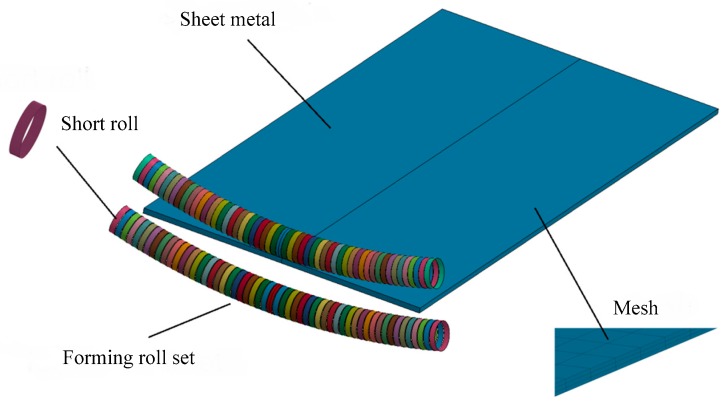
Finite element model based on flexible rolling equipment.

In finite element modeling, the flexible rolling apparatus was simplified into a pair of forming roll sets. The forming roll sets must have the following features: (1) The forming roll sets have enough rigidity to bend and thin the metal plate; (2) the contour of roll set can be configure into various shape curve; and (3) the forming roll sets can rotate about their own axis. The flexible rolling model is shown in Figure 3. The forming roll sets consist of many rigid short rolls, they can be configured in a specific arc, and single discrete roll can rotate around its own center axis at the same time. As it is unnecessary to analyze the force and deformation of a forming tool and it is needed to improve the calculation efficiency and the convergence of the model, each discrete roll is used as rigid-body; the metal plate is used as flexible solid. In the meshing, eight-node hexahedron elements are used. In the thickness direction, three layer meshes are used. The metal plate is moving under friction, the friction coefficient is 0.2. Reduced integration is used in the finite element model, the simulation result with reduced integration has numerical differences, but computing time is much reduced, and it has better practicability and enough reliability. The constraints of the motion boundary conditions should be imposed on the reference point of each rigid body short roll. In this process, the contour of forming roll sets are adjusted to the specific shape; the upper forming roll set drops to the target position, and the lower flexible roll is immobile; after the metal plate is caught by two forming roll sets; the upper and the lower forming roll sets have the same rotation speed and they rotate in opposite directions, the metal plate is fed under the friction, the forming roll sets begin to rotate until the processing is completed. In this paper, the material is 1050 aluminum alloy in numerical simulation, the material properties are shown in Table 2.

## 3. Results and Discussion

### 3.1. Analysis of Simulation Results and Discussion

#### 3.1.1. Analysis of Local Forming Non-Uniform of the Formed Part

The metal plate keeps a linear contact with the forming tools in flexible rolling process, the formed surface part is affected by various factors in the experiment, it is easily caused forming defects. Local forming non-uniform of the formed part with the larger bending deformation is the one of the common forming defects, the utilization ratio of the formed part reduced due to this forming defect.

As the flexible rolling is the process based on uneven thickness reduction in the transverse direction, the steady thickness distribution in the longitudinal direction is a necessary condition for ensuring the forming uniformity of the formed part. In order to study the local forming non-uniform of the formed part, in this numerical simulation experiment, the material is 1050 aluminum alloy, and the metal plate size is 100 mm (width) × 200 mm (length) × 1.8 mm (thickness), the target bending radius is 220 mm, the measuring points are in the axis of width middle.

**Figure 4:**
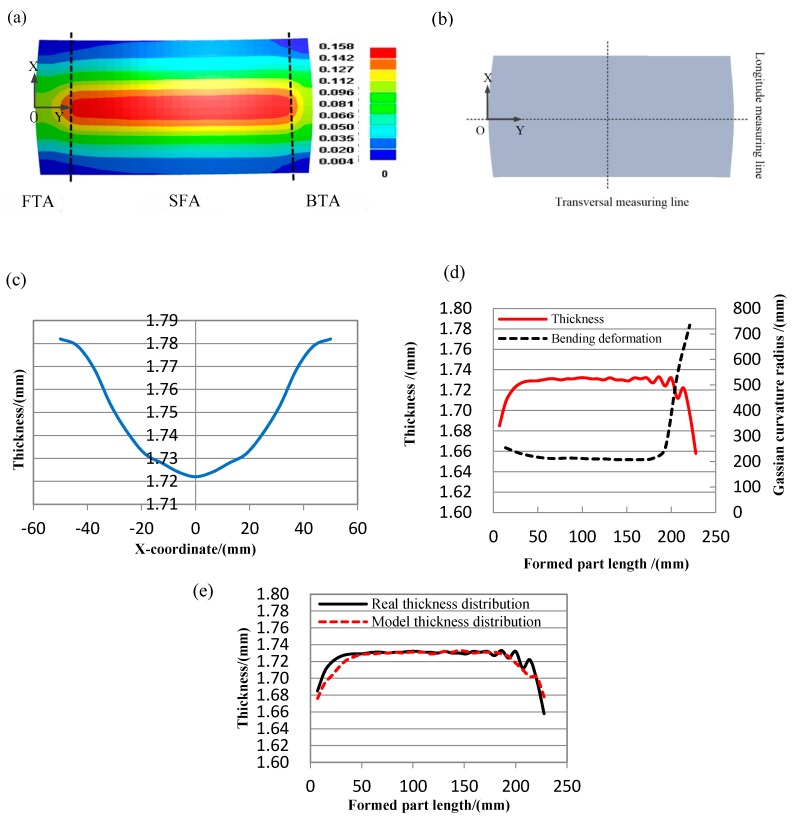
The formed part with (**a**) plastic strain distribution, (**b**) measuring lines in the middle of formed part, (**c**) transversal thickness distribution, (**d**) the local bending deformation and longitude thickness distribution, (**e**) and real thickness distribution and model thickness distribution.

The plastic strain of formed part is shown in Figure 4a. Taking the location in the middle of the formed part as an example in this paper (Figure 4b); in the transversal direction (X direction), the plastic strain is gradient, which is related to the roll gap distribution, the transversal thickness of formed part is decreasing from outside to inside, the transversal thickness distribution curve in the middle of formed part is ideal and caused by the roll gap distribution (Figure 4c); as shown in Figure 4a,d in the longitude direction (Y direction), near the two ends, the plastic strain is gradient as end effect, in this area, the bending deformation is changing, the area is defined as transition forming area (TFA = FTA + BTA); far away from two ends, the plastic strain is stable and continuous, in this area, the bending deformation is stable and continuous, the area is defined as a stable forming area (SFA), it is used as effective area, the utilization ratio is the percent of SFA in the formed part; in the longitudinal direction, the thickness shows a changing trend near both ends of the formed part, while the thickness is continuous and stable far away from both ends, and the thickness distribution corresponds to the bending deformation distribution. As shown in Figure 4e, there are thickness fluctuations near the back end of the longitudinal thickness curve in the middle of formed part with larger bending deformation, a real longitude thickness distribution curve is defective, as shown by the real thickness distribution in Figure 4e; while the target thickness distribution curve should be continuously changing in TFA, and stable in SFA, there are not thickness fluctuations near the back end of the longitudinal thickness curve in the formed part with larger bending deformation, as shown by the model thickness distribution in Figure 4e, it is obtained by numerical simulation. Both in the numerical simulation and forming experiment, the ideal thickness distribution curve of the formed part with larger bending deformation is often obtained in the multi-step forming technology of flexible rolling, a larger bending deformation is divided into a number of small bending deformations in multi-steps forming process, as there are too many steps in this forming technology, so there is low-efficiency in this technology. This forming defect can result in non-uniform bending deformation of the formed part. As the forming defects occur, compared with the formed part with ideal thickness curve, not only the forming uniformity reduced, but also the utilization ratio of formed part reduced.

The reason is that: In the process of flexible rolling, near the back end of the metal plate, the bending deformation greatly reduces due to end effect, the larger bending deformation, the more relative bending reduction, the resistance of material flow suddenly and drastically decreases near the back end, it speeds up the thickness reduction, as a result, the thickness reduction is unstable as sharp decreases, it caused the local forming non-uniform of the formed part.

#### 3.1.2. The Effect of Differential Speed Rotation Technology on the Forming Uniform of the Formed Part

**Figure 5:**
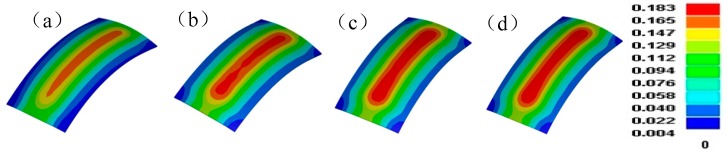
The plastic strain of formed part with different rotation speed ratio (**a**) 1:1, (**b**) 1:1.05, (**c**) 1:1.10, and (**d**) 1:1.15.

**Figure 6:**
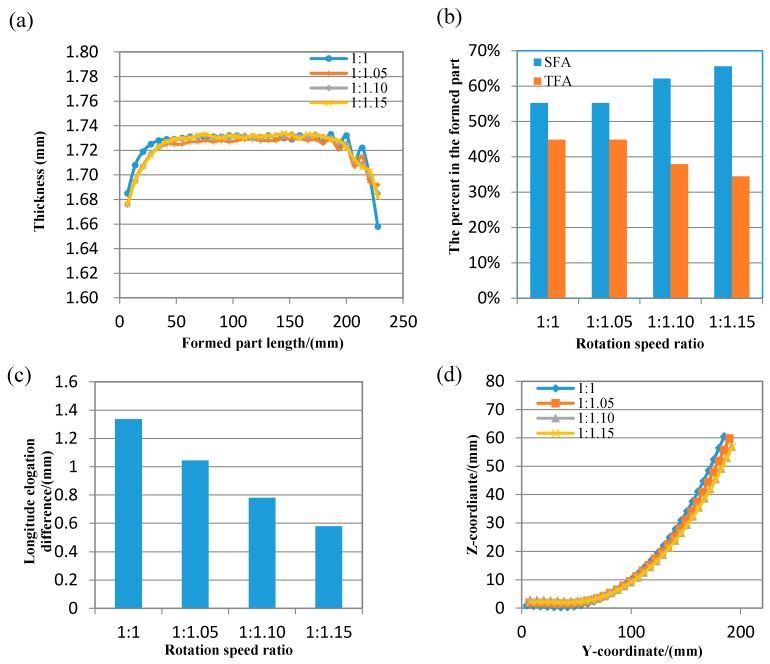
The formed part with different rotation speed ratio (**a**) thickness distribution (**b**) the percent of effective area (**c**) longitude elongation difference between two surfaces (**d**) longitude bending deformation.

In order to improve the local forming non-uniform of the formed part with the larger bending deformation, the differential speed rotation technology is firstly applied. In the compared simulation experiments, the material is 1050 aluminum alloy, the size of metal plate is 100 mm (width) × 200 mm (length) × 1.8 mm (thickness); in order to save computing time of finite element model, the rotation speed in the numerical simulation is much faster than that in the forming experiment; the rotation speed of the upper roll set is 100 mm/s, the rotation speed ratio between upper and lower roll sets is 1:1, 1:1.05, 1:1.10 and 1:1.15 in order, the target curvature radius is 220 mm, the rolling reduction is 0.23 mm; the thickness reduction ratio of all formed parts are about 3.9%. The measuring points are in the axis of width middle, the other processing parameters are same. The plastic strain distributions of formed parts with various rotation speed ratio are shown in Figure 5. The plastic strain is gradient along the transversal direction, in the longitude direction, near the two ends, the plastic strain is gradient as end effect, far away from two ends, and the plastic strain is stable and continuous, the forming effect is satisfied so the differential speed rotation technology is feasible in a flexible rolling process. With the rotation speed difference increases, the plastic strain is increasing. The longitudinal thickness distribution curves of the formed part with various rotation speed ratio are presented in Figure 6a. As the rotation speed difference increases, the fluctuates of longitude thickness curve tend to be steady, so the local forming non-uniform of the formed part is greatly improved in differential speed rotation technology of flexible rolling process. The percent of forming areas in the formed part is shown in Figure 6b, with the rotation speed difference increases, the forming defects are decreasing, the effective area length is increasing, and the utilization ratio of the formed part is increasing. The reason is that: When the rotation speed of upper forming roll set is constant, the rotation speed of lower forming roll set is increasing, the friction between the upper roll set and metal plate is relatively decreasing, and on the contrary, the friction between the lower roll set and metal plate is relatively increasing, the longitude elongation difference between the lower surface and upper surface goes down (Figure 6c), the sharping decreasing of thickness reduction is avoided, thickness relative variation also decreases between successive positions, the continuous stability of thickness distribution is greatly improved, the overall forming uniformity of the formed part is also greatly improved. In the YZ plane, Y direction is the longitude direction, Z direction is the thickness direction, the longitude bending contour curve of formed part is shown in Figure 6d. As the longitude elongation between two surfaces is restrained, the longitude bending deformation also slightly reduces.

### 3.2. Analysis of Experiment Results and Discussion

**Figure 7:**
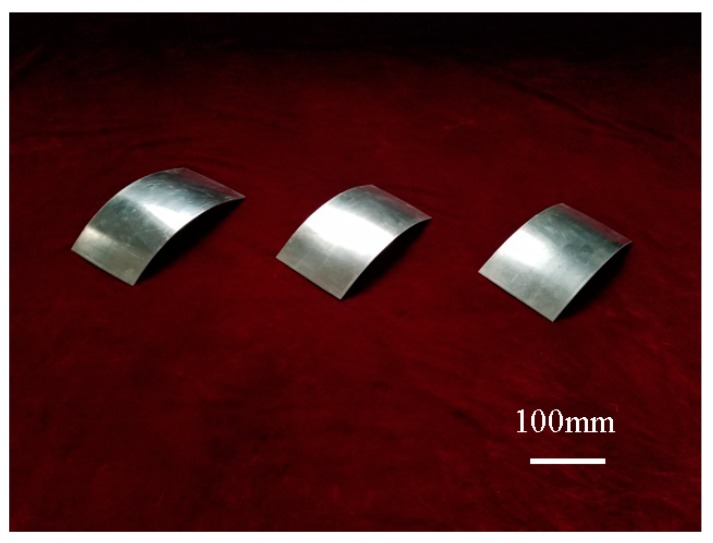
The formed parts with different rotation speed ratio.

**Figure 8:**
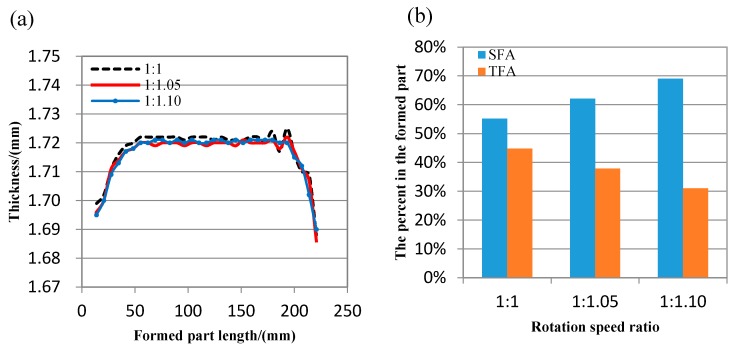
The formed part with different rotation speed ratio (**a**) thickness distribution (**b**) the percent of effective area.

In order to verify the reliability of numerical simulation results, forming experiment is carried by flexible rolling experiment equipment developed by Jilin University. In the forming experiments, the material is 1050 aluminum alloy, the size of metal plate is 100 mm (width) × 200 mm (length) × 1.8 mm (thickness), the rotation speed of the upper roll set is 21.5 mm/s, (it is 100 mm/s in the numerical simulations), the rotation speed ratio between upper and lower roll sets is 1:1, 1: 1.05 and 1:1.10 in order, the target bending radius is 220 mm both in the numerical simulations and forming experiments; the rolling reduction is 0.20 mm in the forming experiments, (it is 0.23 mm in the numerical simulations); compared with the thickness reduction ratio that is about 3.9% in the numerical simulations, it is about 4.4% in the forming experiments; the roll sets contours are unchanged in the forming experiments and numerical simulations. As shown in Figure 7, the formed parts are smooth, and the forming effect is good. The longitudinal thickness distribution curves of the formed part with different rotation speed ratio are shown in Figure 8a. It can be seen that as the rotation speed difference increases, the fluctuates of longitude thickness curve tend to be steady, so the local forming non-uniform of the formed part with large bending deformation is greatly improved in differential speed rotation technology of the flexible rolling process. The percent of forming areas in the formed part is shown in Figure 8b, with the rotation speed difference increases, as the forming defects are decreasing, the effective area length is increasing, and the utilization ratio of formed part is increasing. The result of numerical simulation is in good agreement with the result of forming experiments.

## 4. Conclusions

In this paper, in order to improve the local forming non-uniform of formed part with large bending deformation, the finite element model was established by the flexible rolling forming apparatus developed by Jilin University, the reason for the local forming non-uniform of formed part with large bending deformation was studied; the differential speed rotation technology of flexible rolling process was proposed. Numerical simulated feasibility was finally verified by experimental results.

1. In the process of flexible rolling, as end effect, the bending deformation of the metal plate greatly reduces the thickness reduction suddenly and drastically decreases near the back end, as a result, the thickness reduction is unstable as the sharp decreasing, so it caused the local forming non-uniform of the formed part.
2. With the rotation speed difference increases, the friction between the upper roll set and metal plate is relatively decreasing; the friction between the lower roll set and metal plate is relatively increasing, the longitude elongation difference between the lower surface and upper surface is gone down (Figure 6c), the sharping decreasing of thickness reduction is avoided, thickness relative variation is also decreasing between successive positions, the fluctuates of longitude thickness curve tend to be steady, so the local forming non-uniform of the formed part with large bending deformation is greatly improved in differential speed rotation technology of flexible rolling process, as the forming defects are decreasing, the effective area length is increasing, the utilization ratio is increasing, and the cost of production is decreasing.

## Figures and Tables

**Table 1:** The metrological characteristic of digital instrument.

Range (mm)	Resolution (mm)	Measurement Tolerance (mm)
0–12.7	0.001	±0.002 mm

**Table 2:** Material properties of 1050 aluminum alloy in the numerical simulation.

**Material**	**Density (kg m^−3^)**	**Young’s Modulus (MPa)**
1050 aluminum alloy	2720	76,000
**Poisson’s ratio**	**Yield stress (MPa)**	**Tangent Modulus (MPa)**
0.34	145	25
